# Congenital central hypoventilation syndrome associated with Hirschsprung's Disease: case report and literature review

**DOI:** 10.1016/j.rppede.2015.10.009

**Published:** 2016

**Authors:** Renata Lazari Sandoval, Carlos Moreno Zaconeta, Paulo Roberto Margotto, Maria Teresinha de Oliveira Cardoso, Evely Mirella Santos França, Cristina Touguinha Neves Medina, Talyta Matos Canó, Aline Saliba de Faria

**Affiliations:** a Hospital de Base do Distrito Federal (HBDF), Brasília, DF, Brazil; b Universidade de Brasília (UnB), Brasília, DF, Brazil; c Hospital Materno Infantil de Brasília (HMIB), Brasília, DF, Brazil; d Faculdade de Medicina, Universidade Católica de Brasília (UCB), Brasília, DF, Brazil; e Secretaria de Estado de Saúde do Distrito Federal, Brasília, DF, Brazil

**Keywords:** Congenital central hypoventilation syndrome, Ondine syndrome, Hirschsprung's disease, Haddad syndrome, PHOX2B gene

## Abstract

**Objective:**

To report the case of a newborn with recurrent episodes of apnea, diagnosed with Congenital Central hypoventilation syndrome (CCHS) associated with Hirschsprung's disease (HD), configuring Haddad syndrome.

**Case description:**

Third child born at full-term to a non-consanguineous couple through normal delivery without complications, with appropriate weight and length for gestational age. Soon after birth he started to show bradypnea, bradycardia and cyanosis, being submitted to tracheal intubation and started empiric antibiotic therapy for suspected early neonatal sepsis. During hospitalization in the NICU, he showed difficulty to undergo extubation due to episodes of desaturation during sleep and wakefulness. He had recurrent episodes of hypoglycemia, hyperglycemia, metabolic acidosis, abdominal distension, leukocytosis, increase in C-reactive protein levels, with negative blood cultures and suspected inborn error of metabolism. At 2 months of age he was diagnosed with long-segment Hirschsprung's disease and was submitted to segment resection and colostomy through Hartmann's procedure. A genetic research was performed by polymerase chain reaction for CCHS screening, which showed the mutated allele of PHOX2B gene, confirming the diagnosis.

**Comments:**

This is a rare genetic, autosomal dominant disease, caused by mutation in PHOX2B gene, located in chromosome band 4p12, which results in autonomic nervous system dysfunction. CCHS can also occur with Hirschsprung's disease and tumors derived from the neural crest. There is a correlation between phenotype and genotype, as well as high intrafamilial phenotypic variability. In the neonatal period it can simulate cases of sepsis and inborn errors of metabolism.

## Resumo

**Objetivo:**

Relatar caso de neonato com episódios de apneias recorrentes, diagnosticado com síndrome de hipoventilação central congênita (SHCC) associada à doença de Hirschsprung (DH), o que configurou síndrome de Haddad.

**Descrição do caso:**

Terceiro filho de casal não consanguíneo, nascido a termo, parto normal sem intercorrências, peso e comprimento adequados para idade gestacional. Logo após o nascimento apresentou bradipneia, bradicardia e cianose, foi submetido à intubação orotraqueal e iniciada antibioticoterapia empírica devido à suspeita de sepse neonatal precoce. Durante internação em UTI neonatal evoluiu com dificuldade de extubação devido a episódios de dessaturação durante sono e vigília. Apresentou quadros recorrentes de hipoglicemia, hiperglicemia, acidose metabólica, distensão abdominal, leucocitose, aumento de proteína C reativa, com hemoculturas negativas e suspeita de erro inato do metabolismo. Aos dois meses foi diagnosticada doença de Hirschsprung de segmento longo, foi submetido à ressecção do segmento e colostomia à Hartmann. Feita pesquisa genética por reação em cadeia da polimerase para pesquisa de SHCC, que evidenciou alelo mutado do gene *PHOX2B* e confirmou o diagnóstico.

**Comentários:**

Trata-se de doença genética rara, de herança autossômica dominante, causada por mutação no gene *PHOX2B*, localizado na banda cromossômica 4p12, que resulta em disfunção do sistema nervoso autônomo. A SHCC também pode cursar com doença de Hirschsprung e tumores derivados da crista neural. Há correlação entre fenótipo e genótipo, além de grande variabilidade fenotípica intrafamiliar. No período neonatal pode simular quadros de sepse e erros inatos do metabolismo.

## Introduction

Congenital central hypoventilation syndrome (CCHS) was first described by Robert Mellins et al. in 1970.^1^ It is characterized by central apnea crises due to autonomic nervous system dysfunction.^2^^-^ 5 Central nervous system malformations, as well as lung and heart disease, should be ruled out. Hypoventilation is accentuated during sleep, particularly in the non-REM phase, in which the autonomic control of breathing predominates.^6^ For this reason it was called Ondine's curse syndrome, based on the Norse myth of Ondine (1811) by Friedrich La Motte Fouque, which tells the story of a nymph who gives up immortality to live a human love; however, when she is betrayed, she curses her faithless lover to forget to breathe while sleeping.^7^

In 1978, Gabriel Haddad was the first author to describe the association between CCHS, Hirschsprung's disease and tumors derived from the neural crest, in addition to hypothesize the familial character of the disease.^8^ Approximately 15-20% of cases of CCHS have Hirschsprung's disease; short segment involvement (rectosigmoid) is more common, but long-segment aganglionosis is also described.^4^^,^ 9 ^-^ 11 Tumors derived from the neural crest (neuroblastoma, ganglioneuroma, ganglioneuroblastoma) occur in 5-10% of cases, especially in the first two years of life.^12^

Initially only cases of severely affected newborns were reported in the scientific literature. However, from 1992 on, cohort studies started to be published, which broadened the scope of the syndrome through new evidence such as clinical variants of later onset and autonomic nervous system involvement in other organs, which expands the possibilities of associated clinical manifestations (cardiac arrhythmias, orthostatic hypotension, abnormal pupillary reflex, esophageal dysmotility, diaphoresis, decreased heart rate variability, chronic constipation, excessive drowsiness after use of sedatives and antihistamines). Confirmation of familial recurrence emphasized the genetic component and the broad phenotypic spectrum.^3^^,^ 9 ^-^ 11 ^,^ 13 ^-^ 17

In 2003, *PHOX2B* gene mutations were identified as responsible for CCHS. The *PHOX2B* gene (paired-like homeobox gene), located on chromosome 4p12, encodes a transcription factor responsible for the regulation of genes involved in the development of the autonomic nervous system.^15^ The most frequently found mutation is a polyalanine expansion in exon 3. More than 90% of affected individuals are heterozygous for this mutation. The normal genotype has a sequence of 20 alanines (20/20 genotype). CCHS occurs from four extra alanines in one of the alleles (20/24 genotype). There is a correlation between genotype and phenotype, i.e., the higher the number of alanines, the greater the severity of clinical findings. The presence of a mutation due to polyalanine expansion has been demonstrated by molecular genetic testing by polymerase chain reaction. Other types of mutations (missense, frameshift) may occur and are demonstrated by gene sequencing.^4^^,^ 11 ^,^ 16

The central breathing control depends on chemoreceptors expressed in specialized neurons located in the retrotrapezoid nucleus in the brainstem bulbar region, sensitive to carbon dioxide levels in Cerebrospinal Fluid (CSF).^2^ They are integrated into the neuronal circuitry that stimulates the phrenic nerve and, consequently, promotes diaphragm movement and controls ventilation in order to maintain homeostasis. Retrotrapezoid nucleus neurons (RTNN) express the *PHOX2B* gene. Mice mutated for the gene, with an increase of seven alanines, show 85% reduction of RTNN and can survive only on artificial ventilation.^18^

Most of the mutations responsible for CCHS are de novo mutations. Approximately 10-25% of parents are asymptomatic carriers, responsible for transmission to their offspring.^15^^,^ 16 ^,^ 19 ^,^ 20 Four possibilities are described for asymptomatic carriers: presence of minor polyalanine expansion (up to three, i.e., 20/23 genotype), 20/24 or 20/25 genotypes with incomplete penetrance, somatic or germinal mosaicism.^11^^,^ 15 ^,^ 16 ^,^ 19 ^-^ 21 A risk of recurrence of up to 50% is estimated.^20^ Recently published studies show extreme intrafamilial phenotypic variability, from the elderly individual with sleep apnea, chronic constipation and lack of pupillary response to atropine, to the neonate with CCHS that depends on continuous ventilatory support.^4^^,^ 5 ^,^ 17 ^,^ 21 ^,^ 22

Thus, the aim of this study was to report the case of a neonate with recurrent apnea episodes, diagnosed with congenital central hypoventilation syndrome (CCHS) associated with Hirschsprung's disease, configuring Haddad syndrome.

## Case description

Male neonate, born at term to non-consanguineous parents (gestational age of 39 weeks and six days) through vaginal delivery without complications, classified as appropriate for gestational age (birth weight 3390 g), had an Apgar score of 8 in the 1st minute and 9 in the 5th minute. The mother was multiparous, with 6 pregnancies and no miscarriages, aged 34 years; the pregnancy was uneventful. The newborn presented, in the first hour of life, with bradypnea, bradycardia and cyanosis, underwent endotracheal intubation and antibiotics for suspected early neonatal sepsis. The echocardiography showed a patent foramen ovale without hemodynamic repercussions. The transfontanellar ultrasonography showed no abnormalities suggestive of central nervous system malformations. The patient had delayed meconium elimination, on the 4th day of life and only after rectal stimulation. He was assessed by the staff of pediatric surgery, which identified a circular stenosis at 3.5 cm of the rectum through digital rectal examination.

During hospitalization in the Neonatal ICU, in the first month of life, the patient had difficulty to undergo extubation. Several attempts were made to withdraw mechanical ventilation with the use of noninvasive positive pressure ventilation, but he persisted with desaturation episodes during sleep and wakefulness. Additionally, there were recurrent episodes of metabolic acidosis and three episodes of respiratory acidosis during ambient air maintenance attempts. He also developed hypoglycemic episodes alternating with hyperglycemia, recurrent abdominal distention, leukocytosis and fluctuating increased C-reactive protein levels with negative blood cultures. Inborn errors of metabolism were suspected. The best option to offer nutrition was through continuous gavage, as had episodes of hypoglycemia approximately one hour after feeding. If the glucose infusion rate was increased, he soon showed hyperglycemia.

There was a family history of a brother with a similar neonatal picture, who died at 10 months from respiratory complications, with an undefined etiological diagnosis, but managed as a carrier of organic acidemia (3-hydroxy-3-methylglutaryl-CoA lyase deficiency). Thus, tests were performed to screen for inborn errors of metabolism (expanded neonatal screening test and research of organic acids in the urine) and a leucine, isoleucine and valine-free formula was initiated empirically, associated with the use of high-dose vitamins t (biotin, riboflavin and L-carnitine). There was no clinical improvement after the institution of dietary therapy and megavitamin. The newborn screening test with mass spectrometry and research of urinary organic acids were normal.

At two months, he underwent a barium enema, which showed cone-shaped transition zone at the splenic flexure and dilatation upstream and Hirschsprung's disease was diagnosed. He underwent intestinal biopsy, affected segment resection and Hartmann's colostomy. Anatomopathological examination showed the presence of a nerve plexus, but absence of ganglion cells in the rectum, sigmoid, transition zone and descending colon; nerve plexus with ganglion cells was present only in the transverse and ascending colon.

Given the suspicion of congenital central hypoventilation syndrome (CCHS) associated with Hirschsprung's disease and after ruling out the hypothesis of inborn error of metabolism, genetic testing was performed using the molecular technique of polymerase chain reaction to screen for CCHS.

## Discussion

Congenital central hypoventilation syndrome (CCHS) is a rare, but probably underdiagnosed disorder. In 1999, there were approximately 160-180 known cases worldwide.^5^ The French registry, published in 2005, estimated an incidence of 1:200,000 live births in France.^10^ In 2009, there were 1000 cases confirmed by molecular studies.^3^ In Brazil, case reports of isolated were published.^7^^,^ 22 This case report aims to alert health professionals about the existence of the syndrome, as well as potential confounders.

It was possible to make the diagnosis of CCHS due to a constellation of factors: the possibility of excluding organic acidemia; the previous experience of members of the multidisciplinary neonatal ICU team who hypothesized the presence of CCHS and persisted in their investigation; the finding of Hirschsprung's disease (HD) during evolution; and access to genetic testing. The patient's brother, also possibly affected by the syndrome, did not achieve diagnostic clarification in life. The doubt between the diagnosis of organic acidemia and CCHS persisted during his 10 months of life, mainly because the excretion of organic acids in urine was demonstrated and there was no access to genetic testing at the time. However, after reviewing the case, it is believed that the increase in acid metabolite excretion was related to the patient's critical condition and hypoxia.^23^ Another interesting fact in the brother's history was the report of hypoglycemic episodes, which were also observed in the newborn described herein, alternating with episodes of hyperglycemia. The oscillations in glycemia were initially attributed to the presumed inborn error of metabolism or the adverse effects of the specific diet. However, there are studies showing liability to maintain normal blood glucose in patients with CCHS. Different mechanisms are postulated for the finding and, among them, hyperinsulinism.^24^^,^ 25

The presence of a mutated allele in the *PHOX2B* gene was demonstrated by the molecular technique of polymerase chain reaction, but the technique used does not quantify the number of polyalanine expansions, as it only identifies the presence of above than normal expansions. However, it seems to be the most severe phenotype, due to the onset of symptoms in the neonatal period and the association with Hirschsprung's disease. Approximately 87-100% of the cases caused by non-polyalanine repeat expansion mutations (NPARM) have Hirschsprung's disease.^4^ In cases of mutation due to polyalanine repeat expansion, a lower occurrence of association with Hirschsprung's disease is expected, of about 20%.^4^ Neural crest cell-derived tumors are found in 50% of cases with NPARM mutations and in only 1% of those with mutations due to polyalanine repeat expansion.^3^^,^ 4 ^,^ 11

The parents have not been submitted to mutation screening, but considering the history of familial recurrence, it is likely that one of them is a carrier. There are no reports of other cases in the family. The mother has postural hypotension and chronic constipation, which may represent mild symptoms of autonomic nervous system dysfunction.^3^^,^ 13 ^,^ 14 Approximately 10-25% of cases of CCHS are due to inherited mutation, transmitted by asymptomatic carriers.^20^^,^ 21

Central hypoventilation is the cardinal sign of CCHS and the characteristic with the highest morbimortality.^4^ The genotype is associated with the need for mechanical ventilation. Carriers of the 20/25 genotype have later clinical presentation and are rarely depend on ventilatory support.^4^ On the other hand, carriers of the genotype 20/27 to 20/33 usually depend on continuous ventilatory support.^4^ Mortality is mainly due to sudden death or pulmonary complications secondary to prolonged mechanical ventilation. Many develop pulmonary hypertension, *cor pulmonale* and hypoxic-ischemic lesions, caused by inadequate ventilatory support.^4^^,^ 9 ^,^ 10 ^,^ 13

The diagnosis and management of these patients are quite challenging. The rarity of the disease, the broad differential diagnosis (malformations, sepsis, inborn errors of metabolism), restricted access to specific genetic tests, the need for ventilatory support at birth and follow-up with a multidisciplinary team (pediatricians, intensivists, pediatric surgeons, geneticists, physical therapists, nurses, nutritionists) are the basis of the previous statement. The survival and quality of life can be improved through the planning of early tracheostomy and gastrostomy, efficient deinstitutionalization process, access to home care programs and even the possibility of diaphragmatic pacemaker implantation.^4^^,^ 9
